# How does evidence-based medicine training affect medical students’ views on traditional, complementary, and alternative medicine and the conspiracy theories about COVID-19: a nationwide study

**DOI:** 10.1017/S1463423623000464

**Published:** 2023-11-24

**Authors:** Özlem Coşkun, Yavuz Selim Kıyak, Işıl İrem Budakoğlu

**Affiliations:** Department of Medical Education and Informatics, Gazi University Faculty of Medicine, Ankara, Turkey

**Keywords:** COVID-19, complementary medicine, evidence-based medicine, medical students, public health

## Abstract

**Aim:**

It was aimed to explore the relationship between evidence-based medicine (EBM) training and medical students’ views on traditional, complementary, and alternative medicine (TCAM) and their views on conspiracies about COVID-19.

**Background:**

Medical students constitute the future workforce of primary health care services. The relationship between EBM training and their views on conspiracies about COVID-19 is critical to explore for providing a better primary health care. The relationship EBM training and medical students’ views on TCAM is also important in this regard.

**Methods:**

This is a cross-sectional study. Turkish medical students were surveyed about EBM training, TCAM, and COVID-19 conspiracies. The electronic survey form consisted of five parts: Demographic characteristics, views and self-perceived knowledge about TCAM and the methods, views on the origin of SARS-COV-2, participation in EBM training, and views on TCAM training. A total of 49 medical schools provided response. Along with descriptive statistics, Chi-square test was utilized.

**Findings:**

Among 2577 participants, 24.0% of them believed SARS-COV-2 was artificially designed. The students who have participated in EBM training via both lecture and small group discussions have a less positive view on TCAM than both the students who have not participated in any EBM training (*p* < 0.05) and the students who participated in only-lectures (*p* < 0.05). There was a significant association between EBM training and whether believing COVID-19 (SARS-COV-2) has been designed purposefully by some people or it has emerged naturally *χ* ^2^ (1) = 17.21 *p* < 0.001. The odds of thinking COVID-19 emerged naturally was 1.85 times higher (95% CI: 1.38-2.47) if the students have participated in EBM training via both lectures and small group discussions than if they have not participated in any EBM training. EBM training affects medical students in terms of beliefs on COVID-19 conspiracies.

## Introduction

Evidence-based medicine (EBM) is ‘the conscientious and judicious use of current best evidence from clinical care research in the management of individual patients’ (Sackett *et al.*, 1996). It necessitates physicians to combine the best scientific evidence, their own clinical experience, and patient values. Since the doubling time of medical knowledge is becoming shorter in years (Densen, 2011), teaching EBM to medical students is crucial for their success in lifelong learning and professional life.

Along with choosing the best evidence, patient values also are important for medical decisions. Therefore, the preferences of the community served should be acknowledged and respected by physicians. Traditional, Complementary, and Alternative Medicine (TCAM), which has been commonly used by the public in various countries (Eardley *et al.*, 2012), attracts growing interest from patients all around the world. However, it could be witnessed a negative perception of TCAM among physicians (Veziari *et al.*, 2017). Even if this negative perception could stem from weak scientific evidence on some TCAM methods, it could affect patient behavior. For instance, it has been revealed that more than 60% of headache patients failed to disclose their TCAM use to their conventional doctors (Adams *et al.*, 2013). The negative perception would be due to the fact that TCAM training does not meet the need (Gray *et al.*, 2019). The other cause might be overlooking the patient values component of evidence-based medicine by focusing only on evidence (Kelly *et al.*, 2015) in undergraduate medical education. Along with physicians’, medical students’ attitudes and knowledge toward TCAM have been well studied (Akan *et al.*, 2012; Brown & Bilszta, 2021; Joyce *et al.*, 2016; Tozun *et al.*, 2022). However, the effect of evidence-based medicine training in undergraduate medical education on the attitudes toward TCAM is not well-known.

Another EBM-related topic is conspiracy theories about the origin of COVID-19 (SARS-COV-2). COVID-19 pandemic has been subjected to many conspiracy theories from the very beginning (Douglas, 2021). For instance, a study has found that the percentages of thinking COVID-19 has an artificial origin were 18 and 12 among Turkish and British people, respectively (Salali & Uysal, 2020). Believing conspiracy theories about COVID-19 has been strived by researchers to be explained through revealing its psychological correlates (Alper *et al.*, 2021). Apart from psychological components, there are some cognitive antecedents such as epistemically suspect beliefs and cognitive biases (van Mulukom *et al.*, 2022). Education could have effect on these beliefs. However, not only the rates among medical students but also the effect of EBM training on the medical students’ beliefs on the origin of COVID-19 has not been studied before.

To fill the gaps that we mentioned above, it was aimed to explore how evidence-based medicine training affects the views of medical students toward TCAM and the origin of the COVID-19 pandemic.

This study has two main research questions:

1. Is there a relationship between medical students’ views on the origin of COVID-19 (SARS-COV-2) and participation in EBM training?
2. Is there a relationship between the delivery method of EBM training and the views of medical students on TCAM?

There are three secondary research questions:

1. What are the views of medical students on specific TCAM practices approved by the Ministry of Health (MoH)?
2. What are the views of medical students on common non-TCAM treatment practices that are not approved by the MoH?
3. What are the medical students’ preferences on TCAM training?

## Methods

### Study design

This is a descriptive cross-sectional study.

#### Setting

The study has been conducted in Turkey with medical students. Undergraduate medical education lasts six years in Turkey (Budakoğlu *et al.*, 2020). While there is no national curriculum for TCAM training in undergraduate medical education (UCEP Groups, 2020), teaching EBM skills is among national accreditation standards (Çakmakkaya, 2021a). There are 14 TCAM methods that are recognized by the Ministry of Health of Turkey. These are: Phytotherapy, Acupuncture, Cupping, Leech, Hypnosis, Larva, Ozone, Osteopathy, Apitherapy, Mesotherapy, Homeopathy, Music Therapy, Prolotherapy, and Reflexology (Ministry of Health, 2016). The license to perform these methods is given through training by the Ministry of Health professionals to the physicians who are willing to perform.

### Participants

As of 2017, there were around 75 thousand medical students in Turkey. The preferred method to sample was convenience sampling. There were no eligibility criteria other than being a medical student. To reach medical students all around Turkey, the link of the survey form has been sent via medical faculty members. Our fellow scholars in various medical schools in Turkey have shared the link in their students’ online groups. In order to spread the survey link, we contacted the scholars who have personal connection with us. We asked them to share the link in student groups in their medical schools. We also asked them to spread it to other scholars who they know in other medical schools. A total of 49 medical schools provided response from at least one medical student. The form has been accessible for six months beginning from 20 August 2020.

### Instrument

The survey form was created using Google Forms and consisted of five parts: (1) Demographic characteristics, (2) views and self-perceived knowledge about TCAM and the methods, (3) views on the origin of SARS-COV-2, (4) participation in EBM training, and (5) views on TCAM training.

In the second part, it was provided 14 TCAM methods that are recognized by the Ministry of Health. In addition to that, it was also provided a list of commonly used methods for treatment that are not approved by the MoH: Quantum therapy, bioenergy treatment, cleaning of subconscious, prophetic medicine, reiki, tying a rag to a wish tree, amulet, getting a preacher (hodja) to blow, exorcising, bonesetter. These “treatment methods” were extracted from focus group interviews carried out with medical students. Moreover, the form was evaluated by two medical students to ensure that the questions are interpreted by the students aligned with the meaning intended by us.

In the fourth part, it was provided four options regarding the delivery mode of the EBM training:

1. I did not participate in any EBM training (no-training group),
2. I participated in EBM lectures without any practice or small group discussions (only-lecture group),
3. I participated in both lectures and small group discussions,
4. I do not remember if I participated in any EBM training.

The last group (those who do not remember) was excluded from the analysis of the relationship between delivery mode and views on TCAM and the origin of COVID-19. That part of the analysis has been conducted using the first three groups. The other parts were analyzed without excluding the last group.

### Statistical analysis

The statistical analysis was conducted using SPSS v.22.0 for Windows (Chicago, IL, USA). Descriptive statistics were carried out. Regarding the views on TCAM, out of seven, 1–-3 were classified as negative view, four as neutral, 5–-7 as positive. The percentages belong to these three groups have been reported. Along with that, the percentages about views and self-perceived knowledge levels on TCAM methods were also reported.

Chi-square test was utilized to reveal if there is an association between EBM training and whether believing COVID-19 (SARS-COV-2) has been designed purposefully by some people or it has emerged naturally. It also was utilized for testing the relationship between the delivery mode of EBM training and views on TCAM.

### Ethical considerations

Participation was voluntary. Gazi University Institutional Review Board approved the study (code: 2020-102).

## Results

### Characteristics of the participants

Among all the students 3.4% (*n* = 2577) of them participated in the study. Out of 2577 medical students, 61.0% of them were female, 39.0% were male. Their distribution was *Year-1* 23.6%, *Year-2* 18.2%, *Year-3* 18.2%, *Year-4* 17.7%, *Year-5* 14.6%, *Year-6* 7.6%. The average age was 20.82 ± 2.01 (min: 17, max: 29). A total of 49 medical schools provided response from at least one student (min: 1, max: 365). The medical schools were from Central Anatolia region 41.6%, Marmara region 19.9%, Mediterranean region 11.4%, East and Southeast Anatolia region 10.8%, Black Sea region 8.7%, and Aegean region 7.6%.

Among all participants, 14.9% of them did not participate in any EBM teaching (no-training group), 32.4% participated in EBM lectures without any practice or small group discussions (only-lecture group), 33.2% participated in both lectures and small group discussions, and 19.4% did not remember if they participated in any EBM teaching.

### EBM training and COVID-19

Regarding the beliefs of the students on the origin of COVID-19 (SARS-COV-2); 52.9% (*n* = 1363) indicated it has emerged naturally, and 24.0% (*n* = 618) stated that SARS-COV-2 has been designed purposefully by some unknown people. The proportion of those who state “no idea” about the origin of the virus was 23.1% (*n* = 596).

There was a significant association between EBM training and whether believing COVID-19 (SARS-COV-2) has been designed purposefully by some people or it has emerged naturally *χ* ^2^ (1) = 17.21 *p* < 0.001. Based on the odds ratio, the odds of thinking COVID-19 emerged naturally was 1.85 times higher (95% CI: 1.38-2.47) if the students have participated in EBM training via both lectures and small group discussions than if they have not participated in any EBM training. There was no difference between the no-training and only-lecture groups (p>0.05).

### Views on TCAM

Among 2577 students, 30.9% had negative (1-3 out of 7), 22.3% neutral (4 out of 7), and 46.8% positive (5–7 out of 7) view on TCAM.

Chi-square analysis showed that there was no statistically significant relationship between the delivery of training (no-training, only lecture, both lecture and small group discussions) and view on TCAM (negative, neutral, and positive) (*p* > 0.05).

### Views and self-perceived knowledge levels on specific methods

Table 1 reveals percentages about views and self-perceived knowledge levels on 14 specific TCAM methods that are recognized by the Ministry of Health of Turkey as of 2020. The most known one by the participants was “leech,” while the least known was prolotherapy. The most favored one was acupuncture.

**Table 1.** Medical Students’ Views and Self-Perceived Knowledge Levels on 14 TCAM Methods that are Recognized by the Ministry of Health of Turkey

	Self-perceived knowledge level (%)				Views (%)		
Method	I have never heard of	Have heard of but do not know how it is implemented	I know how it is implemented	I know it detailed	I am against it	It can be performed	No idea
Phytotherapy	28.4	45.4	19.4	6.8	6.9	41.6	51.5
Acupuncture	1.5	18.5	57.5	22.6	8.5	75.4	16.1
Cupping	20.6	11.9	41.5	26.0	19.9	45.4	34.7
Leech	1.6	12.3	56.3	29.8	30.4	50.8	18.9
Hypnosis	1.6	38.7	46.3	13.4	24.2	47.2	28.9
Larva	48.7	32.4	14.6	4.3	20.1	16.5	63.4
Ozone	25.3	43.4	22.2	9.0	7.8	40.2	52.0
Osteopathy	65.5	24.8	7.1	2.6	6.6	13.7	79.7
Apitherapy	75.3	18.2	5.1	1.5	6.7	11.3	82.0
Mesotherapy	60.1	27.0	8.3	4.5	6.4	18.7	74.9
Homeopathy	63.8	21.5	9.2	5.6	10.7	13.9	75.5
Music Therapy	16.2	41.1	31.3	11.4	5.8	65.3	28.9
Prolotherapy	82.0	13.2	3.7	1.0	6.2	9.0	84.8
Reflexology	64.4	22.0	9.4	4.1	5.8	18.9	75.2

### Views on other common methods in Turkey

Table 2 presents the views of medical students on 10 common non-medical or non-TCAM methods for treatment. They were marked as “it can be performed” between 4.9% and 32.8% by students.

**Table 2.** Medical Students’ Views on Other Publicly Common Methods for Treatment in Turkey

	I am against it (%)	It can be performed (%)	No idea (%)
Quantum therapy	19.4	16.3	64.3
Bioenergy treatment	31.0	28.6	40.4
Cleaning of subconscious	35.3	32.8	31.9
Prophetic medicine	22.4	17.8	59.8
Reiki	20.0	11.6	68.4
Tying a rag to a wish tree	87.5	6.4	6.1
Amulet	78.4	12.0	9.7
Getting a preacher (hodja) to blow	74.4	15.6	10.0
Exorcising	86.3	4.9	8.8
Bonesetter	79.7	14.1	6.2

Among 2577 medical students, 58.8% of them have never used any of these methods as a patient. The most used methods were phytotherapy 18.0% (*n* = 465), cupping 10.4% (*n* = 269), bonesetter 8.2% (*n* = 212), and getting a preacher (hodja) to blow 7.5% (*n* = 192).

### TCAM in medical curriculum

The students answered ‘Should TCAM be included in the medical curriculum?’ by choosing one of the five options. ‘Not necessary’ was 28.0%, ‘elective lecture’ was 18.5%, ‘compulsory lecture’ was 7.3%, ‘elective, both lecture and practice” was 35.6%, “compulsory, both lecture and practice” 10.6%.

## Discussion

The main aims of this research were to explore if there is a relationship (a) between participation in EBM training and whether believing COVID-19 emerged naturally and (b) between medical students’ views on TCAM and participation in EBM training.

Concerning the first research question, it was asked medical students’ opinions on the origin of SARS-CoV-2, which is the cause of COVID-19. The proportion of the students who think that SARS-COV-2 has a natural origin was 52.9%, while 24.0% believed it is artificial. These results are similar to the previous study that found 54.0% of the Turkish people think SARS-COV-2 has emerged naturally while 18.0% of them believe it was artificially designed (Salali & Uysal, 2020). Apart from the percentages, our study revealed the association between EBM training and their beliefs. Our findings showed that if the students have participated in EBM training via both lecture and small group discussions, their odds of thinking COVID-19 emerged naturally was 1.85 times higher than the no-training group. Given that one of the first available evidence back in those days showed ‘SARS-CoV-2 is not a laboratory construct or a purposefully manipulated virus’ (Andersen *et al.*, 2020) (even if more recent evidence mentioned different possibilities (Holmes *et al.*, 2021)), it can be suggested that EBM training that necessitates students to actively participate in helped students to use an evidence-based approach toward conspiracies. This result can also be interpreted as evidence that supports the effectiveness of EBM curricula in Turkish medical schools, similar to the previous study conducted in Turkey that showed the EBM curriculum is ‘effective in improving students’ knowledge and skills on EBM (Çakmakkaya, 2021b).

Considering that there was no difference between the no-training and only-lecture groups in terms of believing whether it emerged naturally, it can be interpreted that EBM training comprised of only traditional lectures that do not necessitate active participation of the students has no effect on learning as if it has not been carried out. Therefore, it can be concluded that EBM training should be held through the methods that require active participation as the literature suggests (Straus *et al.*, 2019).

Regarding the second research question, the results showed that there is no relationship between participation in EBM training and views on TCAM. Since focusing only on evidence is a serious problem in practicing EBM (Kelly *et al.*, 2015), it could be expected that EBM training leads to a more negative view about TCAM. However, the current results do not imply the presence of this kind of problem. The doctors who have less negative view about TCAM could lead patients to disclose their medical conditions comfortably, as opposed to current problems on disclosing (Adams *et al.*, 2013). However, it is a fact that there are many confounding factors that affected these results. We have no data about them, such as the effect of medical teachers as role models, their personal beliefs, the culture that they live in, and the effect of social media. Considering all these confounding factors and the previous studies that showed the decline in positive attitude toward TCAM in clinical years among medical students (Akan *et al.*, 2012; Furnham & McGill, 2003; Joyce *et al.*, 2016), we cannot claim that the only cause was EBM training.

Apart from EBM curricula, our study has also findings on TCAM training. We found that 72% of the medical students think that TCAM training should be included in the medical curriculum either elective or compulsory. It is consistent with the findings of the systematic review that “medical students are generally interested in learning more about TCAM” (Joyce *et al.*, 2016). Moreover, it was found about half of them think that TCAM training should include practice rather than only lectures. However, the literature suggests that the integration of TCAM into medical curricula is limited (Joyce *et al.*, 2016).

To our study, among TCAM methods that are recognized by the Ministry of Health, the most favored by medical students was acupuncture (75.4%), similar to previous studies all around the world (Joyce *et al.*, 2016), ranging from 77% to above 90% (Brown & Bilszta, 2021). While the most known was “leech”, the least known method was prolotherapy, which also has been found the same in a recent study in Turkey (Demir-Dora *et al.*, 2020). However, an older study conducted in Turkey showed that the most known methods were herbal treatment, acupuncture, and hypnosis (Akan *et al.*, 2012). This change in years indicates that the views and knowledge levels of medical students are dynamic and affected by various factors.

Regarding the common non-TCAM methods, which are not approved by the Ministry of Health, between 4.9% and 32.8% of the students marked the “it can be performed” option for these scientifically baseless methods. It opens an area for future research to understand why these medical students do not see any objection to implementation of the methods that are clearly not backed by evidence, such as getting a preacher (hodja) to blow, tying a rag to a wish tree, or quantum therapy.

There are some limitations of this study. The first limitation is that the response rate covered only 3.4% of medical students in Turkey, which is not adequate to be representative of all medical students. Moreover, the distribution of responses among the regions did not represent the actual rates well. However, it may be considered a reasonable rate and distribution in nationwide surveys. Another important limitation would be that our study has a self-reported nature and cross-sectional design. Therefore, it is not possible to establish a causal relationship between EBM training and the other variables. Additionally, there were many confounding factors that we were not able to take into account in the survey. Therefore, educational intervention studies with randomization and a control group would provide more valuable results on this topic.

## Conclusions

The prominent findings of this study are that (a) the medical students who have participated in EBM training via both lectures and small group discussions tend to think SARS-COV-2 emerged naturally rather than purposefully designed by some unknown people, compared to the students who either have not participated in any EBM training or have participated in only lectures and (b) there is no relationship between participation in EBM and views on TCAM. These results could indicate that EBM training has effects on medical students in terms of beliefs on COVID-19 conspiracies. Apart from these main conclusions, the results also indicated that medical students generally have positive attitudes toward TCAM practices approved by the Ministry of Health and negative attitudes toward common non-TCAM treatment practices. They are also willing to learn more about TCAM within medical curriculum.
